# The 2013 Chikungunya outbreak in the Caribbean was structured by the network of cultural relationships among islands

**DOI:** 10.1098/rsos.230909

**Published:** 2023-09-13

**Authors:** Carlos J. Dommar, Leonardo López, Richard Paul, Xavier Rodó

**Affiliations:** ^1^ Theoretical and Computational Ecology Group, Centre d’Estudis Avanßats de Blanes CSIC-CEAB, Blanes 17300, Spain; ^2^ CLIMA Climate and Health Program, ISGlobal, Barcelona 08003, Spain; ^3^ Ecology and Emergence of Arthropod-borne Pathogens unit, Institut Pasteur, Université Paris-Cité, Centre National de Recherche Scientifique (CNRS) UMR 2000, Institut National de Recherche pour l’Agriculture, l’Alimentation et l’Environnement (INRAE) USC 1510, 75015 Paris, France; ^4^ Centre National de la Recherche Scientifique (CNRS), Génomique évolutive, modélisation et santé UMR 2000, 75724 Paris Cedex 15, France; ^5^ ICREA, Barcelona, 08010 Catalonia, Spain

**Keywords:** Chikungunya, flight model, mobility network, Caribbean Sea

## Abstract

In 2013, the Caribbean underwent an unprecedented epidemic of Chikungunya that affected 29 islands and mainland territories throughout the Caribbean in the first six months. Analysing the spread of the epidemic among the Caribbean islands, we show that the initial patterns of the epidemic can be explained by a network model based on the flight connections among islands. The network does not follow a random graph model and its topology is likely the product of geo-political relationships that generate increased connectedness among locations sharing the same language. Therefore, the infection propagated preferentially among islands that belong to the same cultural domain, irrespective of their human and vector population densities. Importantly, the flight network topology was also a more important determinant of the disease dynamics than the actual volume of traffic. Finally, the severity of the epidemic was found to depend, in the first instance, on which island was initially infected. This investigation shows how a simple epidemic model coupled with an appropriate human mobility model can reproduce the observed epidemiological dynamics. Also, it sheds light on the design of interventions in the face of the emergence of infections in similar settings of naive subpopulations loosely interconnected by host movement. This study delves into the feasibility of developing models to anticipate the emergence of vector-borne infections, showing the importance of network topology, bringing valuable methods for public health officials when planning control policies. Significance statement: The study shows how a simple epidemic model associated with an appropriate human mobility model can reproduce the observed epidemiological dynamics of the 2014 Chikungunya epidemic in the Caribbean region. This model sheds light on the design of interventions in the face of the emergence of infections in similar settings of naive subpopulations loosely interconnected by the host.

## 1. Introduction

Chikungunya fever is an acute febrile illness caused by Chikungunya virus (CHIKV) and transmitted by *Aedes* spp. mosquitoes. It was first recognized as a human pathogen during the 1950*s* in Africa. Since then, sporadic epidemics have occurred throughout Africa and South and Southeast Asia up until the early 2000*s* [1,2]. In 2004, there was a resurgence of cases reported in many African countries and a large epidemic spanned from Africa to the southwestern Indian Ocean region, India, and Southeast Asia [3]. In late 2013, there was an unprecedented epidemic in the Caribbean following its first introduction into this immunologically naive territory. The explosive nature of its spatial propagation since the first confirmed case was reported in the island of St Martin, resulting in an epidemic that affected 23 countries in the first six months, 35 countries in the first 12 months [4] and by February of 2016 had already produced more than 1.7 million new infections in the Americas in 45 countries [5].

**Figure 1.**
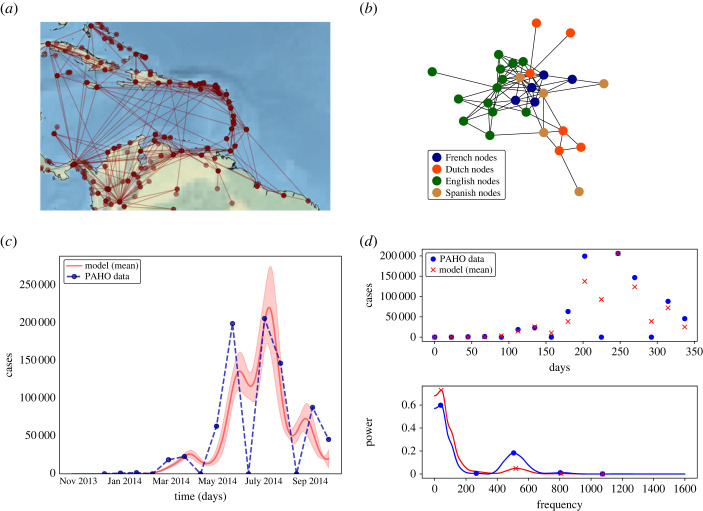
(*a*) Caribbean modelled region and network model based on commercial airline traffic connections during the years 2013–2014 (www.openflights.com [16]). (*b*) Flight network model aggregated by geopolitical entities and colours indicating spoken languages in the simulated locations. (*c*) Aggregated reported Chikungunya cases of the outbreak (blue) and mean model daily solution (red line), the red shadow is the standard deviation for 1000 model evaluations with different parameters from the parameter’s set. (*d*) Weekly reported real case data and model solution (top) and Lomb-scargle periogram for the model aggregated solution and real data (bottom).

The Caribbean outbreak was remarkable not only for its intensity but also for the unpredictable nature of its spread across the myriad of islands comprising the region. Insofar as the islands are principally connected by air travel and given that a significant proportion of CHIKV infections are asymptomatic [6], it is reasonable to assume that infected humans and not mosquitoes spread the virus amongst the islands. Thus, in such a spatially structured epidemic setting where a communicable disease is invading a totally naive population, the geometry of the mobility pattern of the host individuals will probably play a key role. We conjecture that the topology of population mobility is the most important factor driving disease dynamics. In such a context, spatially explicit models are clearly necessary [7,8]. Attempts to model the movement of individuals as a means to understand the rapid propagation of infectious diseases have been numerous and approaches diverse: cellular automata [9], networks [10,11], individual-based models [12] and metapopulation approaches [13]. For spatial spread, both distributed contacts and distributed-infective models have been used with some success [10,14,15]. Here we use a similar approach to that of contact networks to analyse the Caribbean Chikungunya outbreak. Because public health preparedness can enable intervention mitigating against viral invasion, we focused on the first 10 months of the expansion of CHIKV in the Caribbean in 2013 to better understand the main factors determining the initial phase of invasion and propagation (figure 1*a*). This was also selected to avoid potentially confounding effects of within-island herd immunity.

Previous work has introduced the importance of contact networks between human populations in the spread of diseases. Guzzetta *et al.* [17] show the importance of the spatial component in the spreading of Chikungunya in Italy. Zhang *et al.* [18] focused on the continental area and not on the geographical spread throughout the Caribbean island system, which may show a different dynamic due to the isolation of the nodes. The present paper focuses exclusively on the initial stages of the epidemic, avoiding re-seeding stages of the infection in the islands.

Finally, Salje *et al.* [19] assessed the effect that social structures have on rural communities in Bangladesh. Once again, the role of connectivity and contact networks established by these communities was studied, but in a different environment than that represented by island systems, in which the isolation of the nodes makes the network structure a preponderant factor in the overall dynamics of the system.

Thus, the work is structured as follows. Firstly, in the Model formulation section, we proceed to properly describe the compartmentalized model that underlies the node level and the network model that provides the spatial component and how both approaches complement each other. Next, the Results section describes the main results obtained by the model using different topological configurations in the contact network in order to validate the hypothesis of the importance of linguistic grouping in the spread of the outbreak. In the Discussion section, each of the modelled counterfactual scenarios is analysed in detail and contrasted with the real scenario, showing the importance of linguistic clustering and demonstrating that more serious outbreaks could have occurred if the initial conditions of the system had been different. Finally, in the electronic supplementary material, a more detailed analysis of the model is offered, explaining how the main parameters that determine the dynamics were determined; a parameter sensitivity analysis determines the robustness of the method and finally, the model is validated using a different dataset from the original one.

## 2. Material and methods

### 2.1. Model formulation

An epidemic compartmental model built as a set of coupled SEIR (susceptible-exposed-infected-recovered classes) ordinary differential equations (ODEs) was coupled by a host mobility model developed on the basis of a commercial flight network dataset (model (2.1)). This way, the topology of the mobility network that interconnects the equations describing the infection dynamics represents the flight connection network of the region at the time of the outbreak. The flight dataset was obtained from openflights.org [16]. In order to recreate a network that is as realistic as possible to the 2013 context, we use the data from the aforementioned repository (open-flights) corresponding to that year (2013). As a consequence, this topology reflects an approximation of the mobility pattern of people in the region, namely during the epidemic event investigated. The epidemic global SEIR model is structured as a network where each node of the network represents a local population and is modelled as a deterministic local SEIR ODE system. This local node ODE system is in turn coupled with other similar SEIR nodes via migration rates given by the link strengths drawn from the flight network of connections.

2.1 $$\begin{array}{rl} & \frac{dS_{i}}{dt}=-\beta_{i}\frac{S_{i}I_{i}}{N_{i}}+\sum_{j}\tau_{ij}S_{j}-\rho_{i}S_{i},\\ & \frac{dE_{i}}{dt}=\beta_{i}\frac{S_{i}I_{i}}{N_{i}}-\sigma_{i}E_{i}+\sum_{j}\tau_{ij}E_{j}-\rho_{i}E_{i},\\ & \frac{dI_{i}}{dt}=\sigma_{i}E_{i}-\gamma_{i}I_{i}+\sum_{j}\tau_{\;ji}I_{j}-\rho_{i}I_{i},\\ \mathrm{and}\;\;\;\;\; & \frac{dR_{i}}{dt}=\gamma_{i}I_{i}+\sum_{j}\tau_{\;ji}R_{j}-\rho_{i}R_{i}\\ & \;\;i,j=1,\ldots ,n. \end{array}\}$$

The assumptions of the model (2.1) are as follows: (i) Each node can be modelled as a homogeneous SEIR ODE system that represents the local epidemic dynamics of a particular isolated population in an area (island, country, territory, etc.); (ii) Nodes are coupled with other nodes by host migration rates; (iii) The network of flight connections is a good approximation of the pattern of the human host mobility among locations in the Caribbean region; (iv) Mosquitoes are in excess and therefore their population dynamics do not matter for explaining the infection dynamics in the host population. This assumption transforms the vector-borne model into a host-to-host infection process, but with the addition of a transmission rate parameter that integrates mosquito mediated infection as a constant. Under this assumption, the infection term, i.e. the force of infection times the number of susceptible individuals, *λ*S, simply depends on the number of susceptible and infectious hosts, *β*SI, as there are always enough mosquitoes to infect people. Finally S, E, I, R, N are the number of susceptible, exposed, infectious, recovered and total individuals, respectively; and lastly, we assume that (v) mosquito movement between nodes is zero or it can be neglected.

One of the characteristics of the proposed model is that it does not explicitly consider the population of vectors as mentioned above. Vector dynamics play a role in a vector-driven system, but we assume there is a non-limiting mosquito population size, which is realistic in tropical regions. Previous models have been shown to adequately match observed Chikungunya epidemic data in La Réunion and Colombia without the need to complicate the model with vector dynamics for which detailed data are lacking [20,21].

One consequence of the former assumptions is that human hosts are the only carriers involved in the geographical spread of CHIKV in the region. The mathematical description of the SEIR model is given by the ODE system (2.1), where the indices *i*, *j* denote locations and *n* is the total number of nodes. We have then 4*n* coupled equations. S_i_, E_i_, I_i_, R_i_ denote the local population fractions of susceptible, exposed, infected, recovered, N_i_ = S_i_ + E_i_ + I_i_ + R_i_ is the total population of human hosts at each location *i*. We thus assumed in (iv) of the previous model assumption list that the per capita transmission rate integrates the local mosquito dynamics.

These assumptions hypothesize that the main mechanism for the propagation of CHIKV during the outbreak in the Caribbean would essentially be a process driven by the human host mobility carrying the virus to remote locations and that a flight network should capture the spatio-temporal dynamics in the early stages of the outbreak. Additionally, these assumptions permit the implementation of a relatively simple epidemic model with few infection parameters to fit the data—namely the transmission rate of infection *β*, the rate of change from exposed to infection classes *σ*, and the recovery rate *γ*, where *σ*^−1^ and *γ*^−1^ are the incubation period (average time of a typical exposed individual to become infectious) and the duration of infection (i.e. average time of a typical infected individual to recover from the infection), respectively [22] (see table 1). This approach with few parameters to estimate produces a convenient framework for analysis, tractability and especially to facilitate intuition on the qualitative behaviour of the epidemic dynamics. Because the mobility network is relatively stable in time when compared with the time scales of infection spreading, the model can serve as an operational framework to create strategic control policies for the geographical propagation of arboviral infections of loosely connected naive territories.

**Table 1.** Main model parameters. The network-related parameters are explained later in the network model section. *β*, *σ* and *γ* are the common parameters of a classic *SEIR* model and the meaning in this model is well explained above in this section.

parameter	definition
*β*	transmission rate
*σ*	incubation rate
*γ*	recovery rate
*τ*_*i*,*j*_	immigration rate of individuals to node *i* from nodes j=1, …, n
*ρ*_*i*_	emigration rate at which individuals move out from node *i*

Figure 1*b* shows the network model based on reported flight connections and used for coupling at each node the local *SEIR* sub-models. Each node in the network models a location for which data exist on reported incidence of Chikungunya (both confirmed and suspected cases) as reported by PAH Organization [23].

For setting the model’s initial node population sizes, corresponding real inhabitant numbers from censuses for these locations were used [24]. The existence of a link between any two nodes indicates that there is at least one commercial airline operating between them at the time of the outbreak. Each link has an associated *strength*. The link strength between any two locations is proportional to the number of commercial air flights operating between them and, here, it is assumed to be directly proportional to the number of passengers traveling. Countries/Islands represented as local populations by the network nodes are the following: Puerto Rico, Dominican Republic, Jamaica, Haiti, Cuba, Cayman Islands, Bahamas, Colombia, Venezuela, Antigua and Barbuda, Barbados, Dominica, Martinique, Guadeloupe, Grenada, Virgin Islands, Saint Kitts and Nevis, Saint Lucia, Aruba, Bonaire, Curaçao, Sint Eustatius, Sint Maarten, Anguilla, Trinidad and Tobago, British Virgin Islands, Saint Vincent and the Grenadines, Montserrat, Saba and Saint Barthelemy.

## 3. Results

Within 10 months of the first reported case on the island of Saint Martin in October 2013, 4148 Chikungunya cases were reported sequentially in 28 additional islands or mainland territories throughout the Caribbean [23]. The flight connection network of these islands for that time span, along with the number of commercial flight companies between each location, were retrieved from openflights.org [16].

Figure 1*a* shows the flight connection network in the Caribbean and northern South America. Using the observed flight network structure, an epidemic network model was developed (equation set (2.1)). The data provided by openflights.org gather the number of commercial carriers operating between any two airports in the region. These airport connection data were used to develop a human mobility network model. We assume that the numbers of flights are proportional to the number of passengers traveling. In the fitting process, a scaling factor is computed to adjust for this assumption. In the network model, each island is considered a node and these nodes are connected according to the observed flight network topology; the connections or links represent the air transportation paths between the local populations of the islands or mainland territories in the investigated region. All the airports belonging to the same location were grouped into a single node and their respective link strengths were summed. This produces a network model where nodes represent local homogeneous populations and links represent the entire mobility flux that enters or exits each node population. This generates a model of closed subpopulations that are loosely connected by mobility links. Additionally, the epidemic network model was constructed as a set of local susceptible-exposed-infected-recovered (SEIR) population compartmental models, with one SEIR population model for each node of the network. Each SEIR node model assumes a homogeneous contact structure. This renders the spatial resolution of the model structure at the level of nodes. The mathematical description of the model is given by the set of ordinary differential equations (2.1). The nodes represent defined locations, either island or mainland territories that reported Chikungunya cases during the first 10 months, so the model can be fitted to the available incidence data. We computed numerical solutions of the model (2.1) at the level of individual nodes. The numerical solutions represent simulated local outbreaks on each island or mainland territory. The node-level numerical solutions were then aggregated (all local node solutions were summed) into a single global solution. The aggregated solution was in turn parametrized and fitted by a nonlinear least-squares method [25] to the Chikungunya aggregated case incidence data [23]. The data fitted numerical solutions used were produced by equation set (2), where the network epidemic parameters *β*^net^, *σ*^net^, *γ*^net^ were estimated globally for the network (electronic supplementary material, table S1). It is important to note that this parameter set is estimated for the aggregated numerical solution across all local node solutions, and should not be interpreted as if the parameters were describing the epidemic dynamics of an unstructured homogeneous or quasi-homogeneous single population.

The main idea to fit the aggregated data of all islands to the sum of all network individual nodes solutions, rather than fitting each observed population to a particular node model, is that this approach produces a unique parametrization of the SEIR for all the nodes of the network. The estimation of the global parameters *β*^net^, *σ*^net^, *γ*^net^ allows us to focus on the effect of the network structure alone on the dynamics of the infection spread, which is one of our main concerns in this study. This would not be clearly examined if we, instead, would attempt to parameterize individual nodes, producing 29 sets of parameter estimates, one for each possible node. This is because we would be introducing an additional source of heterogeneity in the network model, namely the variation of individual node parameters that may modulate the dynamics at local levels in addition to the effect of network topology alone. Therefore, the epidemiological network estimates, *β*^net^, *σ*^net^, *γ*^net^ should not be used to describe epidemic features of individual nodes or particular populations but to describe the whole network. Doing the former would be falling into the ecological fallacy problem [26].

Figure 1*c* shows the reported aggregated data (dotted, blue) for the first 10 months of the Chikungunya epidemic in the Caribbean in 2013, and the data-fitted numerical aggregated solution (continuous, red) of the SEIR-network model (equations set (2)). Their respective smoothed (Lomb-Scargle) spectra are plotted in the two (*d*) panels, showing how the simulated Chikungunya system correctly captures the main variability features dominating the boundary conditions of the system (figure 1*d*, top). Spectral peaks and their relative strengths are both largely comparable between the data and the simulated model observations (figure 1*d*, bottom). The ability of the model to capture the fundamental variability components is notable given the short timespan of data available for model calibration. The model covers well the slower time scales of variability showing up at around 11–50 weeks, and it is sensitive to variability even at the scale of four weeks. Analogous results for the fit to the TYCHO dataset can be found in electronic supplementary material, figure S2a,b. See electronic supplementary material, table S1 for the obtained parameter values for TYCHO.

**Figure 2.**
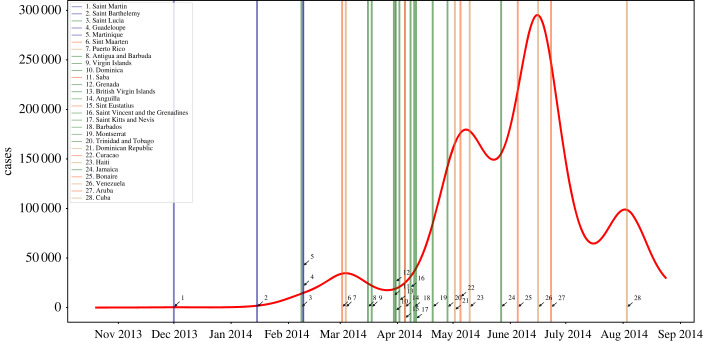
Epidemic wave generated by the aggregated local simulated model solutions at each location or island (node). The simulations were performed using the mean value of the full parameter set (table 2). The vertical blue lines indicate the maximal epidemic peak at each local network node. The legend on the vertical axis denotes the node location as they occurred. The red curve is the aggregated Caribbean model solution.

Figure 2 shows the decomposition of the aggregated epidemic solution into its local island components. Each vertical line marks the time when local epidemics reached their maximum, and it depicts islands and mainland contributions to the global dynamics. Also shown in colour coding is the dominant language spoken on each island. It can be also seen that islands speaking the same language are affected concomitantly by the Chikungunya outbreak. Ignoring local dialects, such as Papieamento and Creole, there are four official spoken languages in the Caribbean region: Spanish, English, Dutch and French [27]. Similar results when using TYCHO can be found in electronic supplementary material, figure S3. Figure 1*b* shows the flight network model where nodes represent islands/mainland territories and colours label nodes grouped by linguistic communities. Based on the conjecture that, for historical reasons, cultural or linguistic related regions tend to be more connected, the structure of the network of the observed commercial flights in the Caribbean would exhibit a topology distinct from that of a random network with no preferential attachment or community structure. If linguistically similar islands or territories were physically more connected by commercial flights, one would expect to observe a higher transit of people among linguistically similar locations. Following this idea, and assuming that the spread between any two locations is carried solely by the movement of infected humans traveling between these two locations, then infectious agents would first spread preferentially within a particular set of islands with a common language (table 2).

**Table 2.** Network metrics for the different model scenarios: (i) observed network, (ii) Saint Martin node with randomized links, (iii) Saint Martin node fully connected with all other nodes and (iv) Erdös–Rényi (E–R) completely randomized network. Metrics for the scenario of Saint Martin with randomized connections, and scenario for E–R were computed and averaged over 5000 random simulations.

network metric	(i) Obs. network	(ii) St Martin + rand. links	(iii) St Martin fully connected	(iv) E–R network
*r*—assortativity Coef.	0.293	0.249	0.167	−0.051
*L*—aver. shortest path	2.19	2.17	1.74	2.43
*C*—aver. clustering	0.492	0.464	0.717	0.1376

To this end, we applied the network epidemic model (2.1) to explore four scenarios where we can alter the flight connection topology by (i) linking the initially infected island, St. Martin, to all the others, or by (ii) randomizing the flight connections of St. Martin. Finally, we also test changes if we were (iii) randomizing all island connections. In a fourth scenario, we change the initially infected island to that being the most important representative node of each language.

In addition to numerically computing the epidemic dynamics of each scenario, we have computed their corresponding Newman’s assortativity coefficient, *r*(*a*). This measures the tendency of nodes of a network to be connected to other nodes that are like them according to some attribute *a* [28]. In our case, the attribute is the particular spoken language in a node (i.e. English, Spanish, French or Dutch). In general, *r* = 0 when there is no assortative mixing, *r* = 1 if there is perfect assortative mixing, i.e. nodes are connected only with those alike according to a given node attribute, and *r* < 0 if the network is disassortative. In general, −1 < *r* ≤ 1 and varying degrees of positive assortativity occur for 0 < *r* ≤ 1 [28,29]. The largest assortativity coefficient computed with respect to the language spoken in the node is that of the observed network when compared with the other three scenarios based on the same number of nodes and links of the observed network—including a pure random network. This supports the assumption that nodes (islands or territories) with the same spoken language tend to be more (flight-wise) connected among themselves than with others (table 3).

**Table 3.** Parameters of the epidemic model obtained by a nonlinear least-squares estimation from the observed incidence Chikungunya series from PAHO. Notice that *β*^net^, *σ*^net^ and *γ*^net^ are network parameters that integrate both the different local epidemic parameters *β*_*i*_, *σ*_*i*_, *γ*_*i*_ where the sub-model nodes are homogeneous and the links strength connecting these sub-models.

parameter	definition	mean fitted value
*β*^net^	network transmission rate	$1.395\;d^{-1}\pm \;0.072$
*σ*^net^	network incubation period	$1.430\;d^{-1}\pm \;0.139$
*γ*^net^	network recovery rate	$1.186\;d^{-1}\pm \;0.068$

Besides the assortativity coefficient, other common metrics to characterize network structure are the average shortest path *L*, and the average clustering coefficient *C*. The average shortest path is the mean number of node-to-node jumps or steps needed to travel along the shortest path between any two nodes over all node pairs of the network [29–31]. The local clustering coefficient of each node can be computed as the proportion of observed triangles or closed triplets over all possible triangles in its neighbourhood. The average clustering coefficient of a network is the mean of individual node clustering coefficients and it gives a measure of the tendency of the nodes of a network to be clustered together [29,32,33]. Table 3 shows the network metrics for (a) the observed flight connection network, (b) the observed network with randomized connections for Saint Martin, (c) the observed network with Saint Martin fully connected to all other nodes of the network and (d) the fully randomized network. The average shortest path length is not defined for disconnected networks. By definition, a network is connected if there are no isolated nodes or sub-network components. Because the E–R algorithm may produce disconnected networks as a result of the random pairing of links and nodes, we computed the average shortest path length for a random selection of 5000 E–R produced random networks that were connected networks.

### 3.1. Network model with initially infected node connected to all other nodes

In this scenario, we set the initial infected node, Saint Martin, to have a connection to all the other nodes in the network while leaving the remaining original topology based on the observed flights. Link strengths (assumed as scaled parameters proportional to the number of flights per unit of time) were assigned to each new connection by randomly picking strength values from the observed network. 5000 simulations were produced this way. Thus, for each simulation, we randomly varied the link strengths while keeping the topology constant.

**Figure 3.**
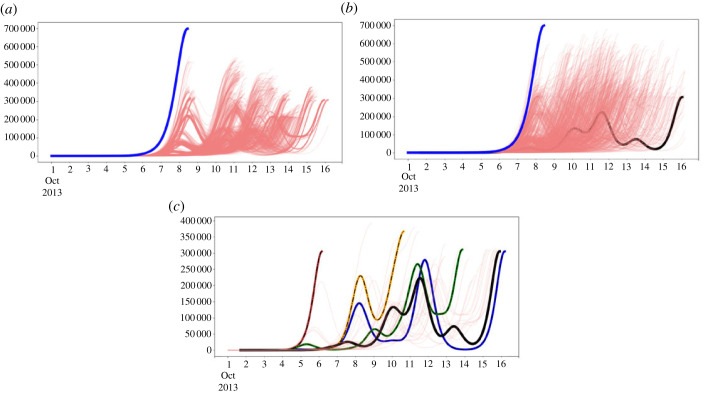
Simulated scenarios. (*a*) Scenario where the links for Saint Martin island were randomized (light red); and (*b*) Scenario where the links for all nodes have been randomized following an E–R random network model (light red). The modelled outbreak initiated in Saint Martin is the black solid curve in all plots. The solid blue curves in (*a*) and (*b*) correspond to the homogeneous model where the initial infected island of Saint Martin is connected to each other node location of the network model. (*c*) Is the number of simulated cases for different outbreak starting locations. The network model integrating airline connections seeded at the most populated location (node) of each cultural setting defined by locations with common spoken language. Hence colours denote cultural compartments, i.e. blue (French), orange (Dutch), brown (Spanish) and green (Anglo/English). Light red curves denote simulations of the fitted model where infection was initiated at each of the nodes of the network model other than Saint Martin (the black curve).

The simulation solutions of this super-connected network scenario are depicted in figure 3*a*,*b* by the solid blue curve. All 5000 trajectory solutions are nearly identically single-peak shaped and appear totally overlapped when plotted. The observation that all solution trajectories seemingly lie on a single curve suggests that the link geometry rather than the distribution of link strengths has the predominant role in determining the spatio-temporal spread of the disease among the local populations at each node. This scenario serves as a benchmark case scenario to assess the role of topology in the dynamics of CHIKV, because by connecting the initial infected node to all other nodes, we essentially eliminate the network structure and make the model a quasi-complete mixed homogeneous SEIR system with no explicit space. Thus, individuals in any node have very similar encounter probabilities with individuals in any other node, independent of their geographical locations.

Wiring the first infected node (Saint Martin Island) to every other node in the network dramatically changes the observed network links and node geometry by reducing the average shortest path length. A smaller average shortest path length means that one can navigate the network from a node to any other node through fewer connected intermediate nodes. The average shortest path length *L* of the network produced by connecting Saint Martin Island to each of the other islands of the network is 1.74, while for the observed network it is much larger, namely 2.19 (table 3). This implies that the contact structure is more closely interconnected in this scenario producing faster disease dynamics that resemble non-spatial homogeneous settings, as can be seen by the solid blue curves in figures 3*a*,*b*.

The assortative coefficient with respect to the node’s spoken language of this scenario case is 0.17, which is significantly smaller than the value 0.29 of the observed network (table 3). This implies that the process of rewiring Saint Martin to all other nodes partially dismantles the language structure of the observed flight connection network. The average clustering *C* for this scenario case is 0.72, which is larger than the observed 0.49 (table 3). This difference is expected because the density of node triangles increases as new links are added to a network with no proportional increase in the number of nodes.

### 3.2. Network model with randomized connections of the initially infected node

In this scenario, the fitted network SEIR model was used to generate simulations when the connections of the initially infected node Saint Martin were totally randomized. The original six links representing flight connections to/from Saint Martin were mingled to form new connections with six other random nodes (excluding self-connection to Saint Martin) along with their corresponding strengths in the observed network. The rest of the links that were not involved in this random perturbation were preserved.

Figure 3*a* shows, in light-red, 5000 random simulations of the aggregated epidemic curve that resulted from implementing this random connection rewiring. For comparison, the model solution for the aggregated cases starting in Saint Martin as observed is plotted with the solid black curve. The solid blue curve corresponds to the previous homogeneous benchmark scenario. All epidemic curves are plotted up to the time when they reach their global maximum. Even though each simulation is random in the sense that the links at the initially infected node are randomly rearranged for each simulation, the SEIR models at each node are deterministic, so that each curve represents a particular deterministic path trajectory of the model solution for the spread of the infection across the network’s nodes.

Disturbing the network by randomly changing the connections of the initial node, even though keeping the same number of connections and preserving the rest of the network intact, can have a profound impact on the course of the epidemic wave. This is shown by the observed variation of the light-red epidemic curves in figure 3*a*. The infection can follow many different paths producing a variety of epidemic geographical diffusion patterns due to a small disturbance in the network structure, i.e. the links of the initial infected node. As can be seen, the epidemic duration of the outbreak produced by the model based on the observed flight connections (black curve) lies towards the tail of all the simulation distributions of this scenario. This implies that the actual observed epidemic is atypically slower, reaching its maximum later when compared to most other possible epidemic trajectories. Thus, the epidemic dynamics are sensitive to small changes and perturbations in the topology of the network mobility, and the actual observed outbreak is seemingly one of the slowest outbreak outcomes. Assessing this outcome may have profound implications in terms of public health, which we later discuss.

The average assortative coefficient *r* of the networks with regard to language is 0.25 compared with the observed 0.29 (table 3). This shows, as one would expect, that the random rewiring of Saint Martin breaks apart the language structure of the observed flight network. Notably, the randomization of the links of only one node in the observed network is sufficient to produce a less assortative network structure. On the other hand, the average shortest path *L* for networks in this scenario is comparable to the observed network, with values of 2.17 and 2.19, respectively. Similarly, the average clustering *C* of the networks in this scenario and the observed network are comparable with values of 0.46 and 0.49, respectively (table 3), with at most a decrease of the clustering effect by the random rewiring of Saint Martin.

Finally, figure 3*a* shows different heat-maps for these assumptions. Under the assumptions made in this scenario, outbreaks reach their maximum peaks in around 300 days for most of the network nodes. This behaviour has to do with the number of people in the population who are exposed to first-degree risk. This first-order risk affects the time it takes an epidemic to reach the peak of outbreaks. For this particular scenario, the time to reach the epidemic peaks seems quite variable in comparison to the subsequent ones.

### 3.3. Fully randomized E–R network

In this scenario (figure 3*b*, light red curves), we investigate how a network with the same number of nodes and links as the observed network, but built instead by a process that theoretically produces a fully random set of connections between the nodes, would impact on the epidemic trajectory. The algorithm used to completely randomize the observed flight connection model is that of 3 [29]. An uncorrelated random E–R *G*(*n*, *p*) network is a graph where *n* is the number of nodes and *p* is the probability of link formation so *n* nodes are connected through *l* edges which are chosen randomly from *n*(*n* − 1)/2 all possible configurations. Every pair of nodes is connected with probability *p*. The total number of edges is a random variable with an expected value *np*(*n* − 1)/2 hence *p* = 2*l*/(*n*(*n* − 1)). Thus, introducing the observed number of nodes and observed number of links as *n* and *l* in the expression *p*, we obtained the necessary probability of connection to generate a comparable fully random network from the number of nodes of links in the observed network. For details, see [29,34]. Any deviation or difference in the spatio-temporal pattern dynamics of the epidemic between an E–R network and the observed flight network is an indication that the latter is not the consequence of a random process when connecting nodes, but that there is some underlying intended or deliberate reassortment process. This scenario serves also as a null network model when studying fundamental features of the observed network.

Figure 3*b* shows the aggregated epidemic curves of 5000 simulations (in light red) of a fully randomized network based on both the number of links and nodes of the observed model of flight connections. While the number of links is kept the same as in observations, they are rearranged in a random manner according to the E–R algorithm. Outbreaks are always shown up to the maximum peak. The black curve corresponds to the epidemic dynamics of the model fitted to the observed data. In comparison to the observed outbreak, the outbreaks now simulated on the fully randomized network exhibit a reduced overall epidemic length. Only five simulations out of 5000 had longer durations than the observed network in our random sample. The simulated epidemics are all growing generally earlier and faster than the observed epidemic trajectory.

The average assortativity coefficient based on language was *r* = −0.0507 compared with *r* = 0.29 of the observed network (table 3). This difference reveals that the full randomization of the observed network completely eliminates the language structure of the observed network, transforming it into a network with no assortative mixing. The average shortest path length was *L* = 2.43 and the average clustering was *C* = 0.14 (table 3). By normalizing these values against their respective values in the observed situation (observed/ER), their significance can be weighted. Normalized quantities $\hat{L}=0.902$ and $\hat{C}=3.58$ indicate that the average path length is similar in both the null full random network model and the observed flight network, but the clustering of the observed one is more than 3.5 times higher than that of the fully random model. This difference again indicates that the clustering structure of the observed network is not a product of chance alone and that conversely, it may have a small-world type of structure, a feature reported in many social and man-made networks [29,34,35]. The positive assortativity coefficient of the observed flight network regarding the language of the island further supports this conjecture and suggests that cultural and political factors are influenced by the particular spoken language of the island. These factors likely have a prominent role in the formation and maintenance of the topology of the current flight connection, and hence also in the human mobility patterns in the region.

Given that the average shortest paths are similar in both the observed network and the fully randomized E–R network but that the observed network has a higher clustering, one would expect to see at least a similar speed of the epidemic in both cases or even a faster outbreak dynamic in the observed network. Therefore, the observation that the actual observed epidemics show a relatively slow evolution suggests that the initial condition, i.e. the start of the epidemic in the node of Saint Martin, has had a significant role in slowing the outbreak. Should the epidemic have started in some other location, we would likely have observed a much faster progression of the disease in the region. As expected and as can be seen in figure 3*c*, under the assumptions made in this scenario, the rate of spread of outbreaks seems to slow down overall compared to the previous scenario. The outbreaks reach their maximum peaks at a time close to 250 days for most of the network nodes and very high dispersion of the results exist, which is a product of the topology changes. This slowdown may again be linked to the number of people in the population exposed to first-degree risk, affecting the time it takes to reach the peak of the outbreaks.

### 3.4. Network with the initial infection started at each of the different network linguistic nodes

In this scenario, we address the significance of the island where the epidemic started on the resulting regional epidemics. Therefore, we simulate the outbreak from each of the nodes of the observed network. The outbreak trajectories starting in distinct islands are represented by the light-red curves shown in figure 3*c*. The simulated outbreaks coloured in blue, green, yellow-orange and brown show simulations that started in the most populated locations of the four linguistic groups that dominate the Caribbean region. That is, the blue curve is the outbreak that starts in Haiti (French-speaking location); the green curve is the outbreak starting in Jamaica (English), the yellow-orange outbreak starts in Curaçao (Dutch) and the brown outbreak starts in Colombia (Spanish). As with the previous scenarios, the outbreaks are depicted until they reach the largest peak and thus the epidemic severity is described as the height of this largest peak.

We observe that for this scenario, epidemic trajectories starting at distinct nodes retain the epidemic patterns observed in the outbreak produced by the epidemic starting in Saint Martin, namely the multi-peak curves of aggregated cases, thereby indicating a heterogeneous spatial epidemic wave. However, the speed and magnitude of the epidemic trajectories vary significantly. Since the topology of the network is unmodified and only the initial infected node is varied, this scenario reveals that the observed topology regarding the starting location of the infection has a central role in the spatio-temporal spread of the epidemic in the first stages of the outbreak.

Electronic supplementary material, figure S1 shows the times at which the local epidemic peaks (i.e. the maximum number of infecteds) are achieved at each node for distinct starting points of the infection (a selection of 12 different nodes are shown in the figure), for the fitted model and the observed connection matrix. The colour indicates to which linguistic group the node belongs.

The sequences display how the epidemic evolution would have looked if the initial infected seed was located somewhere other than Saint Martin. These sequences predict how the geographical progression of the infection would have taken place. The pattern of coloured bars suggests a correlation in the order of appearance of the infection with regard to the spoken language in the islands. This spatio-temporal correlation seems to be especially noticeable for French (blue bars) and English (green bars) nodes, supporting the hypothesis that the linguistic aspect of each island in the Caribbean designed the structure of the flight connection and therefore drove Chikungunya epidemics in the Caribbean. Figure 3*c* shows heat-maps for the configuration proposed in this scenario. Under the assumptions made in this scenario, the outbreaks reach their maximum peaks at a time that is above 300 days for most of the network nodes. Again, this change in the initial conditions of the network has a direct effect on the number of people in the population exposed to first-degree risk, affecting the time it takes to reach the peak of the outbreaks.

### 3.5. Impact of scenarios on the geographical spread of the Chikungunya epidemic

Electronic supplementary material, figure S2 shows the cumulative and normalized number of Chikungunya cases (with respect to the total number of cases) as the epidemic spreads through the Caribbean region. The observed cases (first row), the simulated cases of the fitted model (second row) and the average numbers of cases from the two flight network randomized model scenarios (namely, the randomized connections to Saint Martin and the fully randomized E–R) are mapped (third and fourth rows, respectively).

We can see in electronic supplementary material, figure S2 that the temporally spatially scattered diffusion pattern of the observed data is captured by the fitted model and to a lesser extent by the scenario of the initial node with randomized rewiring of its connections. The last scenario, the E–R random network, shows a less scattered structure in the geographical spread of the epidemic, and the pattern is instead more similar to that of a homogeneous spatial model with a smoother and more continuous diffusion among the islands.

### 3.6. Impact of the flight network and language clustering in the dynamics of the outbreak

Finally, in this section, we discuss the relationship between the structure of the contact network and the linguistic grouping with the dynamics of the outbreak.

The first scenario we considered was the homogeneous connection of the initially infected node to all nodes in the network; this scenario served as (i) a benchmark test to evaluate the role of topology in the dynamics, since it is homologous to an unstructured, spatially homogeneous epidemic model and (ii), to assess the importance of the strength of link connections, at least at the scales observed in the network of flights reported for the Caribbean in 2013–2014, and for the dynamics of the initial phase of an outbreak.

As discussed earlier, connecting the initially infected node on Saint Martin Island to every other node in the network drastically changes the observed network connections and node geometry by reducing the average length of the shortest path. In terms of network structure, this means that the outbreak can spread faster than in the real network topology because the contact structure in this scenario is more tightly connected, resulting in faster disease dynamics that resemble a non-spatial homogeneous environment. This scenario serves as a benchmark scenario to evaluate the role of topology in CHIKV dynamics, as by connecting the initially infected node to all other nodes, we essentially eliminate network structure and make the model a quasi-complete mixed homogeneous SEIR system without explicit space. Thus, individuals in each node have very similar encounter probabilities with individuals in every other node, regardless of their geographic location. On the other hand, if we set up the model with random connections of the initially infected node, all epidemic curves are plotted up to the time when they reach their global maximum. Although each simulation is random in the sense that the connections of the initially infected node are randomly rearranged at each simulation, the SEIR models are deterministic at each node, so that each curve represents a particular deterministic path trajectory of the model solution for the spread of infection across the nodes of the network. Thus, since only six links of the original infected node of Saint Martin are randomly rewired to other 29 − 1 = 28 nodes, the rest of the network remains unchanged. It is then possible to compute the number of possible epidemic trajectories as a combinatorial number $\left(\begin{array}{c} 28\\ 6 \end{array}\right)=28!/(6!(28-6)!)$, i.e. 376 740 possible combinations and thus as many different epidemic waves are possible. Owing to computational constraints, we randomly selected and simulated a sample of 5000 of these possible trajectories, confident that this is a representative sample of all possible combinations. The simulated sample represents about 1.3% of all possible trajectories. Disturbing the network by randomly changing the connections of the initial node, even if the number of connections remains the same and the rest of the network remains intact, can have a profound effect on the course of the epidemic wave (figure 3*a*).

Completely random networks with exponential degree distributions, such as those generated by the E–R algorithm, have smaller average shortest path lengths than corresponding non-random networks, which are a mixture of structured and completely random topologies, and skewed degree distributions [29,34].

If we hypothesize that the observed flight link network was not generated as a completely random network because common sense dictates that the establishment of flight links does not follow random rules, but that decisions to establish flight links are anything but random, then we would expect qualitative differences between epidemics generated by completely random networks and the observed network based on flight patterns. Accordingly, we expect completely random networks with exponential degree distributions, such as those generated by the E–R algorithm, to have an average shortest path length that is shorter than these path lengths found in natural networks with a more organized topology that has a mixture of structured and random link geometries and skewed degree distributions [29,34].

This would explain the significantly shorter epidemic duration of the fully random model compared with the observed model. The shortest average paths of the fully random networks are more efficient in spreading the disease in the network and lead to faster outbreaks with shorter extinction times and larger epidemic peaks (figure 3*b*).

Finally, the importance of the network structure becomes clear when comparing 3*a*,*b*. In the first case, the network topology remains similar by simply varying the connection of the initial node, and the dynamics of the outbreaks resemble the real one. The aggregation by cultural nodes is clear, as shown by the results in table S2, where there is a clear aggregation. When the network topology moves away from the real scenario, the aggregation also disappears and the propagation resembles more of a wave (electronic supplementary material, figure S1).

## 4. Discussion

Population spatial sub-structure is known to have a significant impact on infectious disease dynamics, but the relative contribution of sub-population connectedness has remained largely theoretical [36,37]. Here, using the large Chikungunya epidemic in the Caribbean as an example, we show that network topology rather than the strength of network connectedness is the crucial determinant in governing infectious disease propagation, at least during the initial phases of the epidemic. We also reveal the importance of language assortativity and show that the epidemic could have been even more explosive if it had started on another island belonging to a different linguistic domain.

The absence of any impact on the volume of air traffic has been noted before, with the suggestion that maritime transport rather than air traffic was the major contributing factor [38]. While this may indeed be relevant for islands in close proximity, maritime transport is likely to be of little relevance across the greater Caribbean domain. One potential explanation for the reduced impact of air traffic volume is the relatively small probability of a person travelling being infected, especially during the initial phases of the epidemic when the number of cases was relatively small. While individuals with symptomatic Chikungunya would be highly unlikely to travel, asymptomatic CHIKV infections occur albeit with a very variable frequency, ranging from 5 to 80% [6,39,40]. Nevertheless, although asymptomatic or incubating CHIKV infections could lead to inter-island transportation and thus be amplified by the overall traffic volume, it is possible that their number is too low and the variation in traffic too small to be able to detect an impact of overall traffic volume.

The chronology of islands affected clearly shows a linguistic component for both the French and English-speaking sets of islands. Starting slowly in Saint Martin, the next four islands affected were all French-speaking. Remarkably, the northern part of Saint Martin (French), Saint Maarten (Dutch), only declared cases six weeks after Chikungunya occurrence in Saint Martin, suggesting an important structuring effect dominating even at the within island level. The first cases in an English-speaking setting occurred in the islands of Antigua and the Virgin Islands. Subsequently, the epidemic rapidly burnt through all the surrounding English-speaking islands. The significance of language is also suggested by the late occurrence of Chikungunya in Dominica, which lies between the French-speaking islands of Guadeloupe and Martinique, but which only had cases three weeks later. Similarly, Chikungunya cases were signaled at an interval of two weeks in Haiti and the Dominican Republic, both located on the island of Hispaniola. In contrast to the clustering observed in the French and English-speaking islands, the Dutch and Spanish-speaking territories were significantly less clustered in time. It is notable that overall, the largest islands and territories reached their peaks later. This is to be expected as there are more susceptible individuals to infect and thus the peak is reached later. This would explain, at least to some extent, why the Spanish and Dutch islands and territories are less clustered in time.

The persistence of new and emerging infections in the American continent over the subsequent years was presumably attributable to vast numbers of immunologically naive populations along the continent. This is in stark contrast to the Caribbean islands, with more limited populations that are likely to have acquired herd immunity and thus less likely to temporally sustain CHIKV transmission (PAHO). In 2020, however, there were several cases of travelers arriving in metropolitan France having voyaged in the French Caribbean. This would suggest continued transmission in the Caribbean, potentially being persistently re-introduced from the American mainland, likely acting as a large reservoir feeding the Caribbean region (Santè Publique France). For other naive settings worldwide, it is of extreme relevance to focus on the very first stages of the epidemic propagation, as later containment may be absolutely impractical, not to speak about the costly and often ineffective massive mosquito eradication campaigns [41]. Our study delves into the feasibility of developing powerful operational models to anticipate the emergence of vector-borne infections based on simple network mobility patterns that can be obtained from public airport available statistics. Our modelling framework correctly incorporated the temporal and spatial scales of variability in population disease dynamics, using a relatively simple compartmental framework that we applied to a sub-network of the complex Caribbean region. Despite this simplification, the reduced system of 29 islands can effectively incorporate the main spatial drivers of the regional dynamics of spread during the Chikungunya epidemic. This could be further extended to land transportation infrastructure, where road development increases connectivity [42]. The information gained could therefore be used as guidance and serve public health officials to more effectively intervene and mitigate developing epidemics and thereby improve potential containment measures in naive geographical areas. Indeed, conversely, to other diseases and settings [43], transmission at the microscale would not appear to be of particular relevance towards the establishment of an alert system for the entire Caribbean region. The complex simulation of social dynamics in spatial networks [44] appears in this case dramatically simplified by the more dominating cultural framework. This is seemingly the main modulating factor in the CHIKV epidemic spread. Thus, in practical terms, finer-scale high-resolution surveillance and control systems would likely add little extra benefit despite coming at a much greater cost.

Limitations of this work are that our network simulations assumed a static framework persists—the connections have remained constant over time. As the turnover of connections is slow or very slow relative to the time scale of the pathogen spatial spread, we can assume there was little or no change in the network during the epidemic phase of infection studied here. Further progress of this work may incorporate recent advances in mobile phone technology and GPS location. This may enable us to more accurately track the movement of people in real-time, something that would allow us, in the face of a severe epidemic, to build more comprehensive networks for such arboviral diseases, and also to track any changes occurring in the network structure [45]. In any case, the fact that very similar conclusions can be obtained with two different databases of Chikungunya incidences in the region (e.g. PAHO and TYCHO), yields further support to the conclusions of this study.

In conclusion, our study illustrates that people’s movement among islands through flight connections was a fundamental infection mechanism in the epidemiological setting of the Chikungunya outbreak in the Caribbean that started in late 2013. Flight connections among islands come as a natural mobility model. We found that, unlike a homogeneous spatially unstructured model, a simple SEIR system coupled to a network model derived from the observed flight connection pattern in the region captured well the global epidemic curve at these initial stages. We identified that the network had a structure that is far from a purely random structured network, with higher clustering and positive assortativity with respect to the language spoken at each node. Network topology is a crucial determinant in the early phases of the epidemic, and this has important consequences for public health preparedness insofar as the network topology can be known prior to the arrival of any infectious agent and thus the disease dynamics may become more predictable. Additionally, network structure through clustering and language assortativity can enable the simplification of prediction through the simulation of infection dynamics by reducing the complexity of the associated models. This in turn enables better planning of intervention and management strategies based on network topology.

## Data Availability

All the datasets used can be downloaded from the repository: https://github.com/LeonardoL87/ChikungunyaDataset2013. The model’s codes are available in this Github repository: https://github.com/LeonardoL87/ModeloDengue. Supplementary material is available online [46].
